# Supplementary material for: Omega-3 fatty acid epoxides produced by PAF-AH2 in mast cells regulate pulmonary vascular remodeling
Source: Nat Commun. 2022 May 31;13:3013. doi: 10.1038/s41467-022-30621-z (PMC9156667; doi:10.1038/s41467-022-30621-z)
Supplement: Supplementary file 1 — Supplementary information [file 41467_2022_30621_MOESM1_ESM.pdf]

Omega-3 fatty acid epoxides produced by PAF-AH2 in mast cells regulate pulmonary vascular remodeling

SUPPLEMENTARY INFORMATION

Supplementary Figure 1

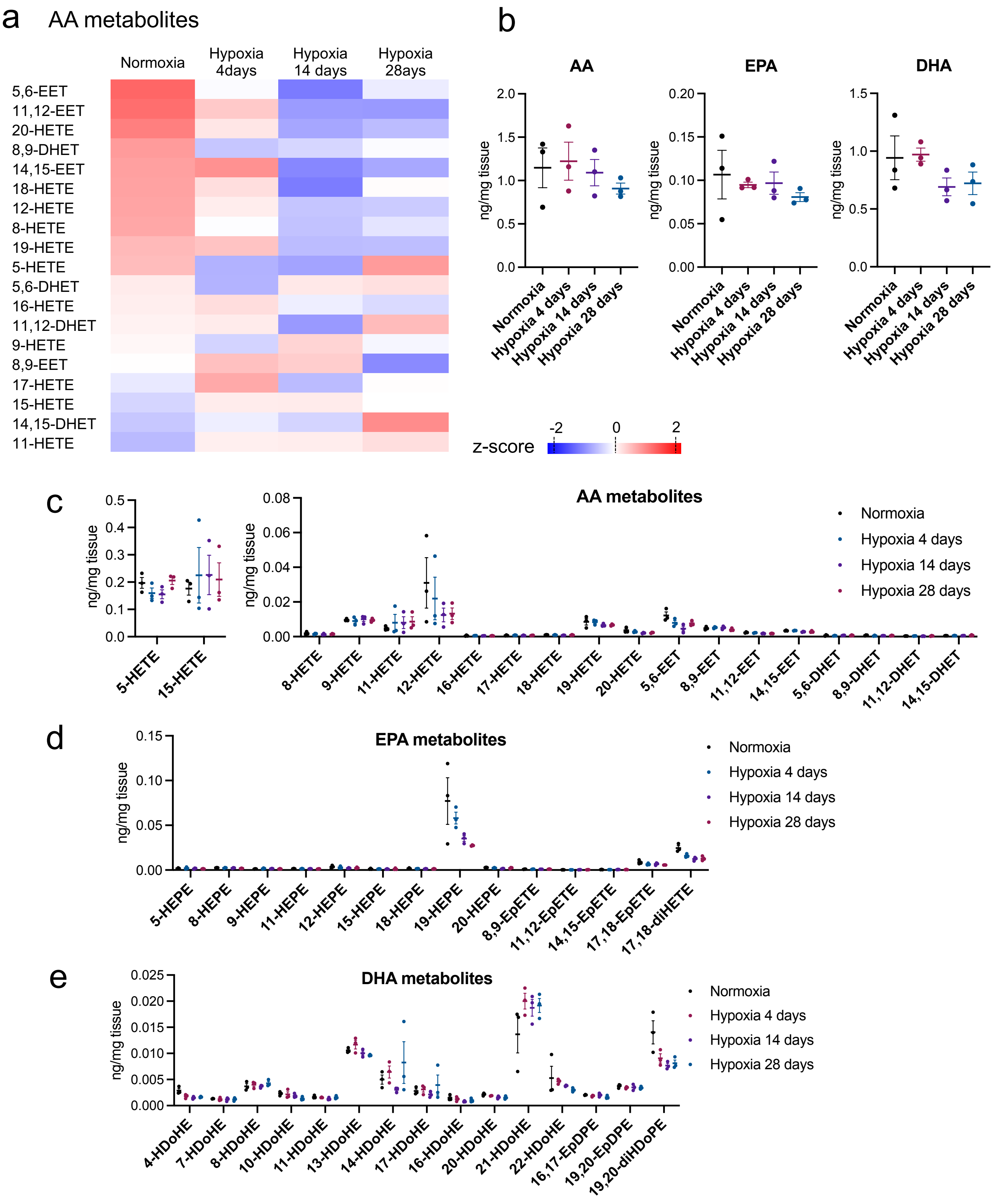

Supplementary Figure 1. (a) AA metabolites in LC-MS/MS based-lipidomics of the lungs of WT mice exposed to normoxia or hypoxia (10% O_2) for 4, 14 and 28 days. Z-score was calculated from the average value of each group (n=3) and shown as heatmap. **(b)** The contents of AA, EPA and DHA assessed by LC-MS/MS based-lipidomics of the lungs of WT mice subjected to normoxia and hypoxia for 4, 14 and 28 days (n=3). **(c-e)** The contents of fatty acid metabolites assessed by LC-MS/MS based-lipidomics of the lungs of WT mice subjected to normoxia or hypoxia for 4, 14 and 28 days (n=3). AA metabolites (c), EPA metabolites (d), DHA metabolites (e). Data are mean ± SEM.

Supplementary Figure 2

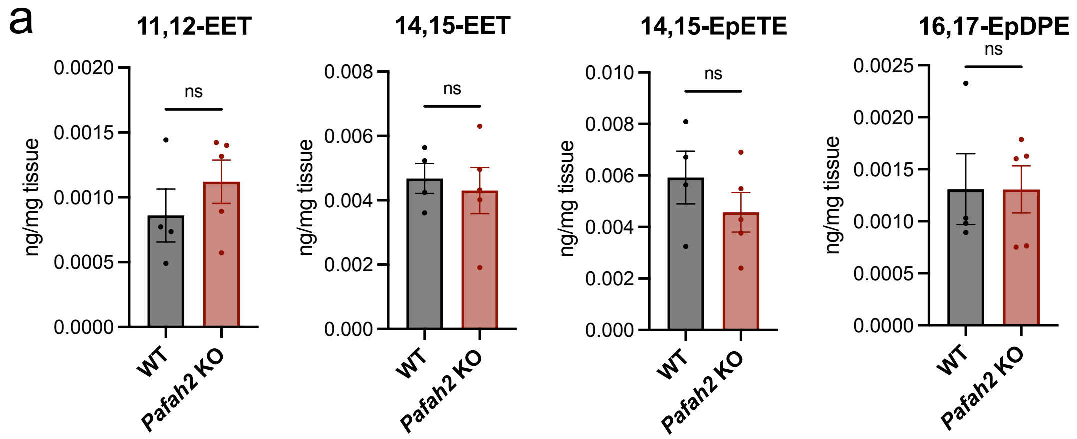

Supplementary Figure 2. (a) The contents of epoxy metabolites assessed by LC-MS/MS based-lipidomics of the lungs in WT mice and *Pafah2* KO mice exposed to hypoxia for 8 weeks (n=4,5). Data are mean ± SEM; ns indicates not significant, by 2-tailed Student's T test.

Supplementary Figure 3

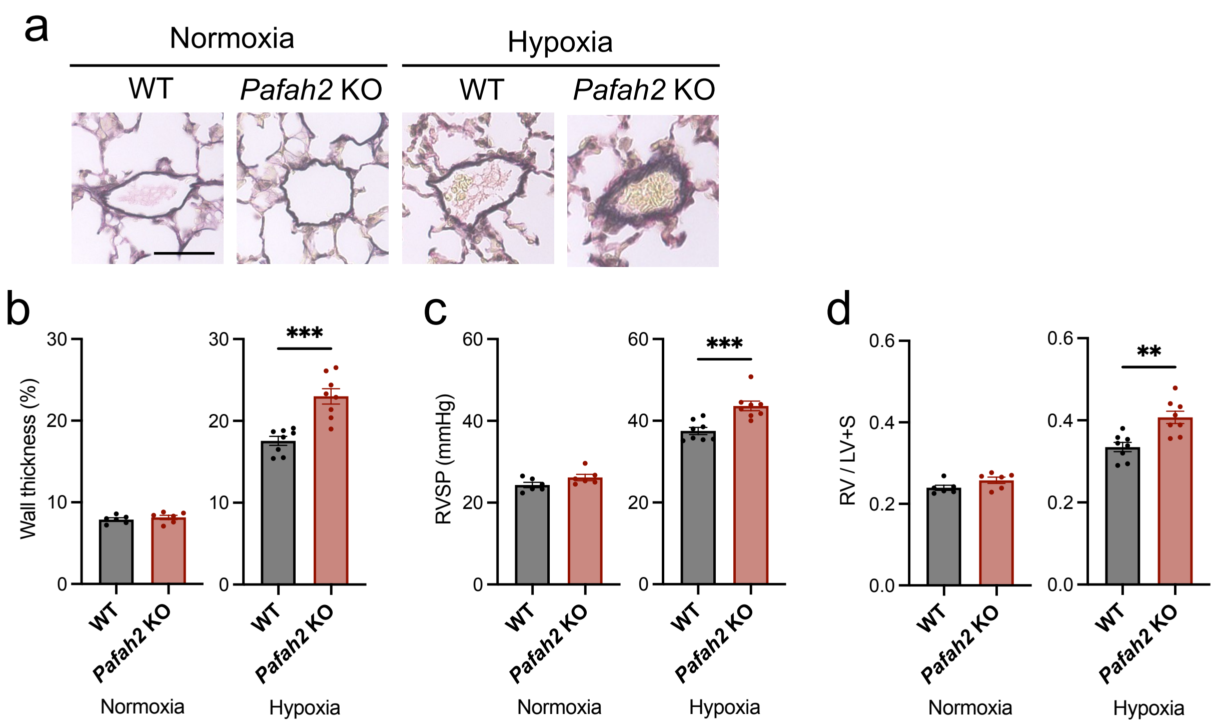

Supplementary Figure 3. (a) Representative image of histological sections with EVG staining (bottom) of lungs in WT and *Pafah2* KO female mice exposed to normoxia or hypoxia for 4 weeks. Scale bar, 50µm. **(b-d)** The evaluation of PH severity in WT and *Pafah2* KO female mice exposed to normoxia or hypoxia for 4 weeks (normoxia, n=6; hypoxia, n=8). Wall thickness of pulmonary arterioles (b), RVSP (c), weight ratio of RV to LV+septum (d). ** *P*=0.0017; *** *P*=0.0002 (panel **b**) and *P*=0.0008 (panel **c**). Data are mean ± SEM. *P* values were determined by 2-tailed Student's T test.

Supplementary Figure 4

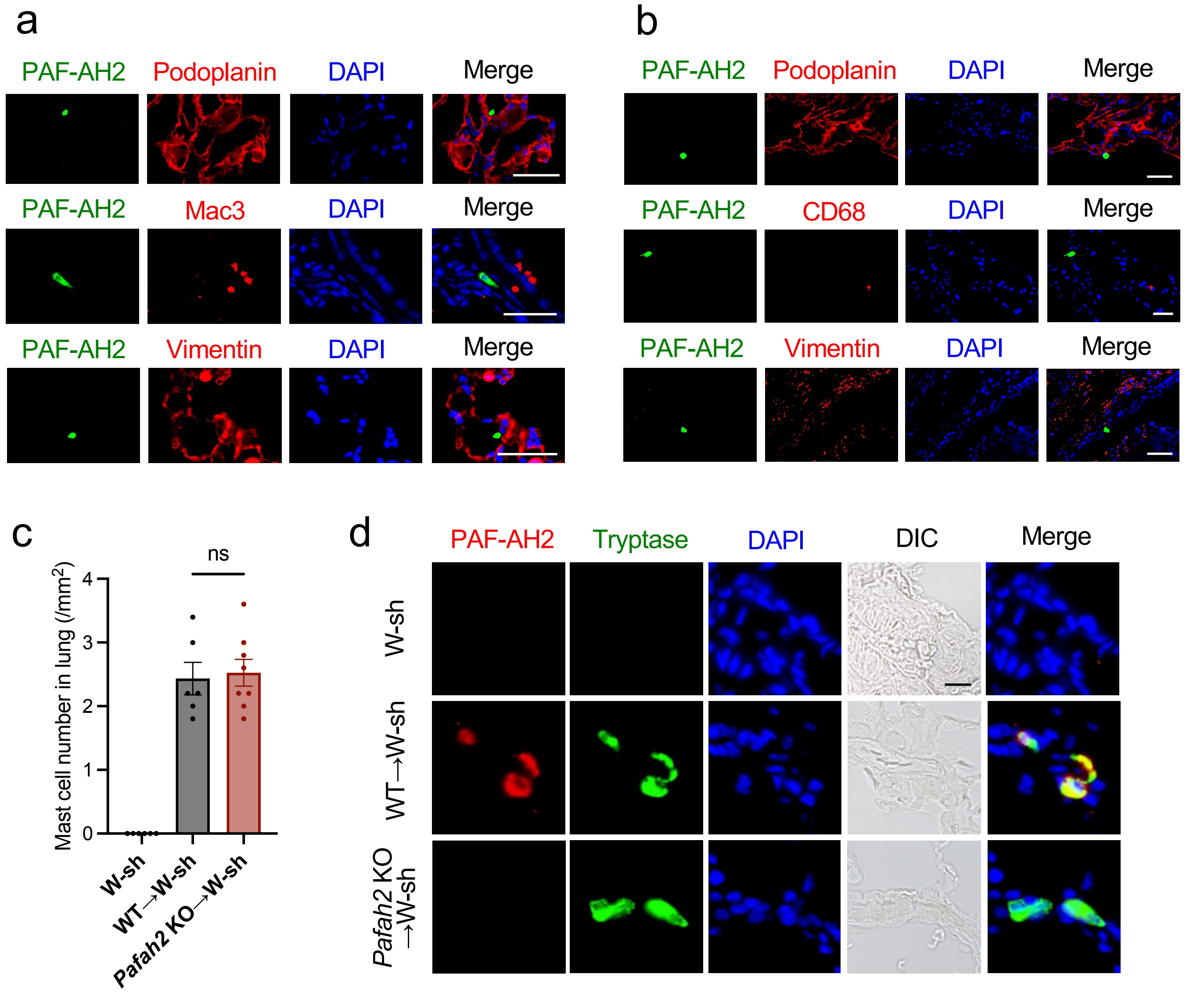

Supplementary Figure 4. **(a)** Representative image of immunohistochemistry of PAF-AH2 and Podoplanin, Mac3 or Vimentin in lung from WT mice exposed to hypoxia for 8 weeks. Nuclei were labeled with DAPI. Scale bar, 50µm. **(b)** Representative image of immunohistochemistry of PAF-AH2 and Podoplanin, CD68 or Vimentin in lung from PAH patient. Nuclei were labeled with DAPI. Scale bar, 50µm. **(c)** Number of mast cells in *Kit* $^{W\text{-}sh/W\text{-}sh}$ mice (W-sh) and those reconstituted with WT BMMCs (WT→W-sh) or *Pafah2* KO BMMCs (*Pafah2* KO→W-sh) evaluated by histological sections with toluidine blue staining (n=6,6,8). Data are mean ± SEM; ns indicates not significant, by one-way ANOVA with Tukey's *post hoc* test. **(d)** Representative image of immunohistochemistry of PAF-AH2 and Tryptase in lung from *Kit* $^{W\text{-}sh/W\text{-}sh}$ mice (W-sh) and those reconstituted with WT BMMCs (WT→W-sh) or *Pafah2* KO BMMCs (*Pafah2* KO→W-sh). Nuclei were labeled with DAPI. Scale bar, 10µm.

Supplementary Figure 5

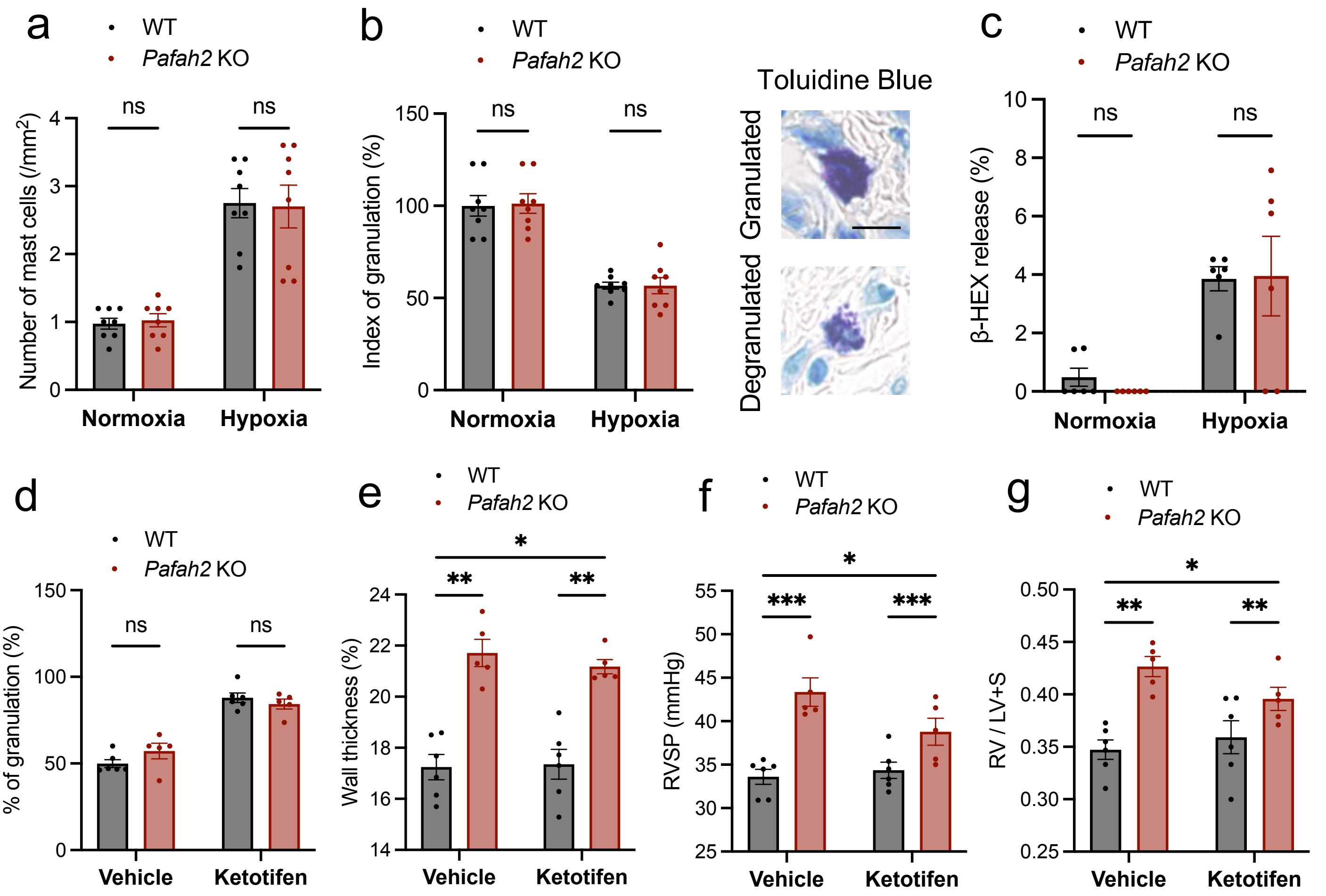

Supplementary Figure 5. (a) Number of mast cells in lungs of WT and *Pafah2* KO mice exposed to normoxia or hypoxia for 4 weeks evaluated by histological sections with toluidine blue staining (n=8). **(b)** Index of granulation (number of granulated mast cells/number of degranulated mast cells) in lungs of WT and *Pafah2* KO mice exposed to normoxia or hypoxia for 4 weeks evaluated by histological sections with toluidine blue staining (left)(n=8). The index of granulation was expressed in percentage assuming that the average in WT mice with normoxia were 100%. Representative figures of histological sections with toluidine blue staining for granulated and degranulated mast cells (right). Scale bar, 10μm. **(c)** β-HEX release of WT BMMCs or *Pafah2* KO BMMCs exposed to normoxia or hypoxia (1% O_2) for 24 hours (n=6). Data are representative of 2 independent experimental replicates. **(d)** Ratio of mast cell granulation (number of granulated mast cells/total number of mast cells x100%) in lungs of hypoxia-exposed WT mice and *Pafah2* KO mice when administered vehicle or Ketotifen (1 mg/kg/day) evaluated by histological lung sections with toluidine blue staining (n=6,5,6,5). **(e-g)** The evaluation of PH severity in hypoxia-exposed WT mice and *Pafah2* KO mice when administered vehicle or Ketotifen (n=6,5,6,5). Wall thickness of pulmonary arterioles (e), RVSP (f), weight ratio of RV to LV+septum (g). * *P*=0.0175 (panel **e**), *P*=0.0467 (panel **f**), and *P*=0.0500 (panel **g**); ** *P*=0.0051 (Vehicle and Ketotifen in panel **e**), *P*=0.0011 (Vehicle in panel **g**), and *P*=0.0085 (Ketotifen in panel **g**); *** *P*=0.0002 (Vehicle and Ketotifen in panel **f**). Data are mean ± SEM. ns indicates not significant. *P* values were determined by two-way ANOVA with Tukey's *post hoc* test.

Supplementary Figure 6

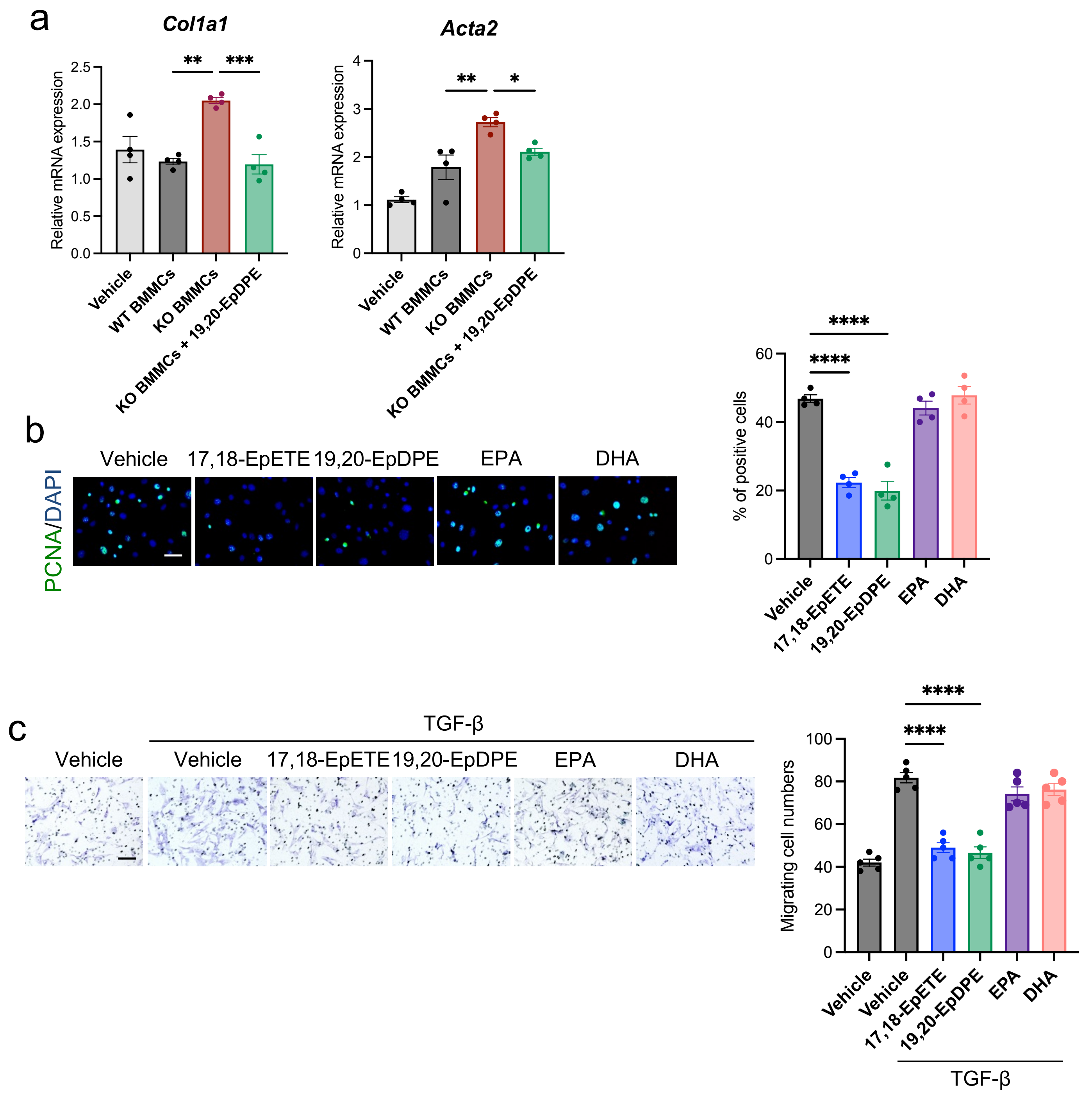

Supplementary Figure 6. (a) Relative mRNA levels of *Col1a1* and *Acta-2* in lung fibroblasts stimulated by lipid extracts when treated with or without ω-3 epoxides (1μM) for 6 hours (n=4). Expression levels were normalized to those of 18S ribosomal RNA and then to those in the fibroblasts treated with vehicle. Data are representative of 2 independent experimental replicates. * *P*=0.0436; ** *P*=0.0013 (*Col1a1*) and *P*=0.0028 (*Acta2*); *** *P*=0.0009. **(b)** Immunocytochemistry for PCNA in lung fibroblasts when treated with vehicle, 17,18-EpETE (1μM), 19,20-EpDPE (1μM), EPA (1μM), or DHA (1μM) for 24 hours (left). Scale bar, 50μm. The percentage of PCNA-positive lung fibroblasts in total DAPI-positive cells (right) (n=4). Data are representative of 2 independent experimental replicates. **** *P*<0.0001. **(c)** Boyden chamber-based cell migration assay of lung fibroblasts stimulated by TGF-β (2.5 ng/ml) when treated with vehicle, 17,18-EpETE (1μM), 19,20-EpDPE (1μM), EPA (1μM), or DHA (1μM) for 24 hours. Toluidine blue staining images (left). Scale bar, 100μm. Migrating cell numbers of lung fibroblasts (n=5) (right). Data are representative of 2 independent experimental replicates. **** *P*<0.0001. Data are mean ± SEM. *P* values were determined by one-way ANOVA with Tukey's *post hoc* test.

Supplementary Figure 7

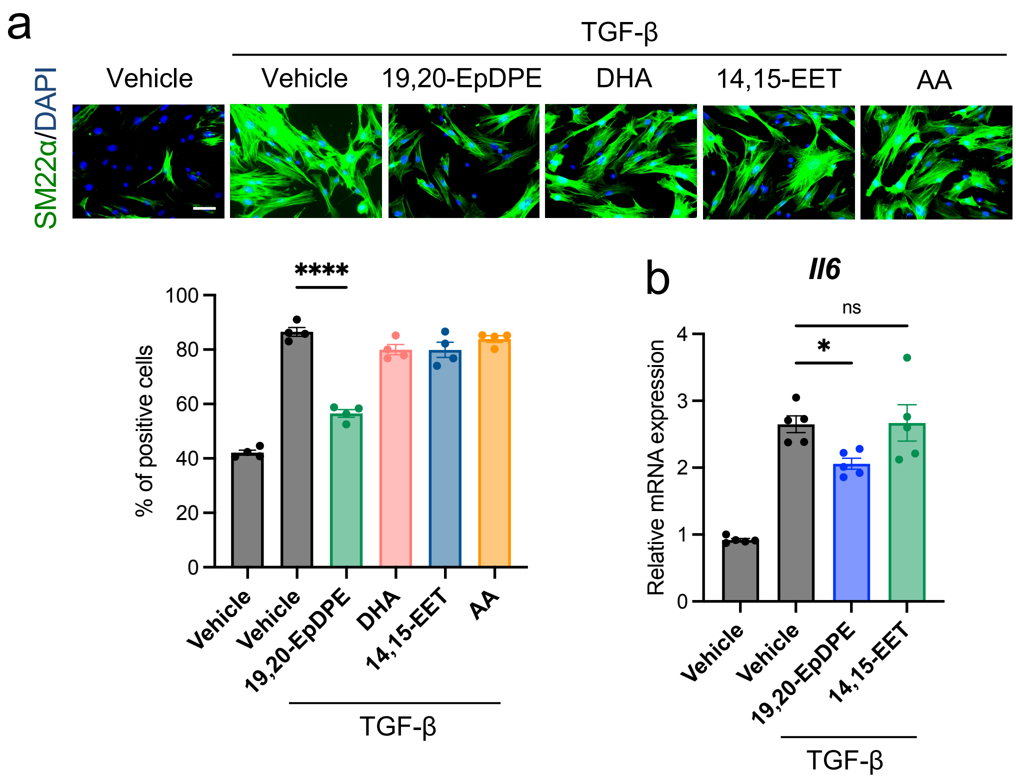

Supplementary Figure 7. (a) Immunostaining of SM22α in lung fibroblasts stimulated with TGF-β (2.5 ng/ml) when treated with vehicle, 19,20-EpDPE (1μM), DHA (1μM), 14,15-EET (1μM) or AA (1μM) for 24 hours (left). Scale bar, 50μm. Ratio of SM22α-positive cells to total lung fibroblasts (right) (n=4). **** *P*<0.0001. **(b)** Relative expression levels of *Il6* mRNA in lung fibroblasts stimulated with TGF-β (2.5 ng/ml) when treated with vehicle, 19,20-EpDPE (1μM) or 14,15-EET (1μM) for 6 hours when (n=5). Expression levels were normalized to those of 18S ribosomal RNA and then to those in the unstimulated control fibroblasts. Data are representative of 2 independent experimental replicates. * *P*=0.0156. Data are mean ± SEM. ns indicates not significant. *P* values were determined by one-way ANOVA with Dunnett's *post hoc* test.

Supplementary Figure 8

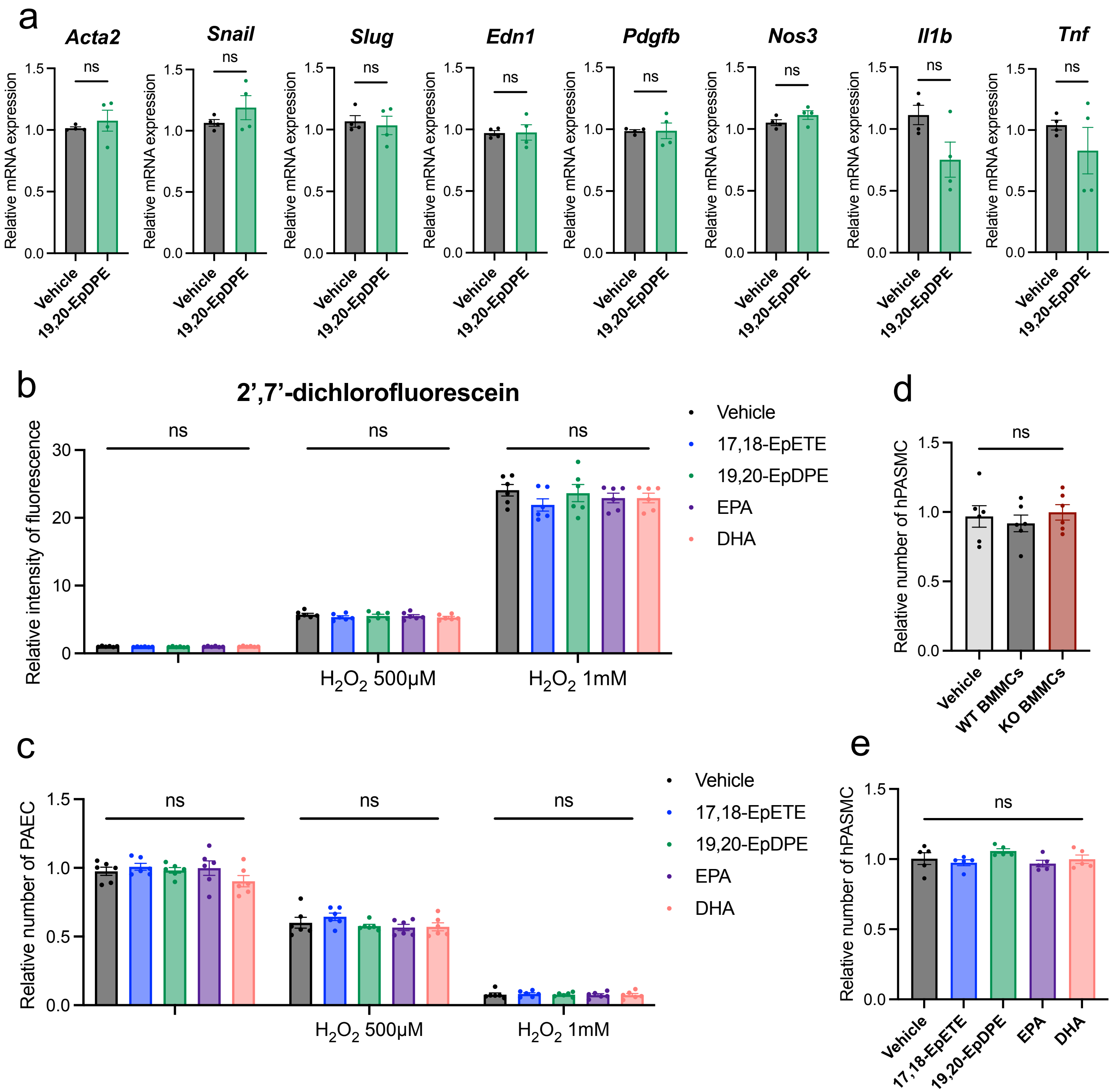

Supplementary Figure 8. (a) Relative mRNA levels of *Acta2*, *Snail*, *Slug*, *Edn1*, *Pdgfb*, *Nos3*, *Il1b,* and *Tnf* in human pulmonary artery endothelial cells stimulated by TGF-β (2.5ng/ml) when treated with vehicle or 19,20-EpDPE (1μM) for 6 hours (n=4). Expression levels were normalized to those of 18S ribosomal RNA and then to those in the endothelial cells treated with vehicle. Data are representative of 2 independent experimental replicates. **(b)** Relative levels of 2',7' -dichlorofluorescein for the detection of intracellular reactive oxygen species in human pulmonary artery endothelial cells stimulated with or without H_2O_2 (500μM or 1mM) when treated with vehicle, 17,18-EpETE (1μM), 19,20-EpDPE (1μM), EPA (1μM), or DHA (1μM) for an hour (n=6). Data are representative of 3 independent experimental replicates. **(c)** Relative number of pulmonary artery endothelial cells (PAEC) stimulated with or without H_2O_2 (500μM or 1mM) when treated with vehicle, 17,18-EpETE (1μM), 19,20-EpDPE (1μM), EPA (1μM), or DHA (1μM) for 2 hours (n=6). Data are representative of 3 independent experimental replicates. **(d)** Relative number of human pulmonary artery smooth muscle cells (hPASMC) stimulated by lipid extracts from cultured medium of BMMCs for 48 hours (n=4). Data are representative of 2 independent experimental replicates. **(e)** Relative number of human pulmonary artery smooth muscle cells (hPASMC) when treated with vehicle, 17,18-EpETE (1μM), 19,20-EpDPE (1μM), EPA (1μM), or DHA (1μM) for 48 hours (n=5). Data are representative of 3 independent experimental replicates. Data are mean ± SEM; ns indicates not significant, by 2-tailed Student's T test (a), two-way ANOVA with Dunnett's *post hoc* test (b,c), or one-way ANOVA with Dunnett's *post hoc* test (d,e).

Supplementary Figure 9

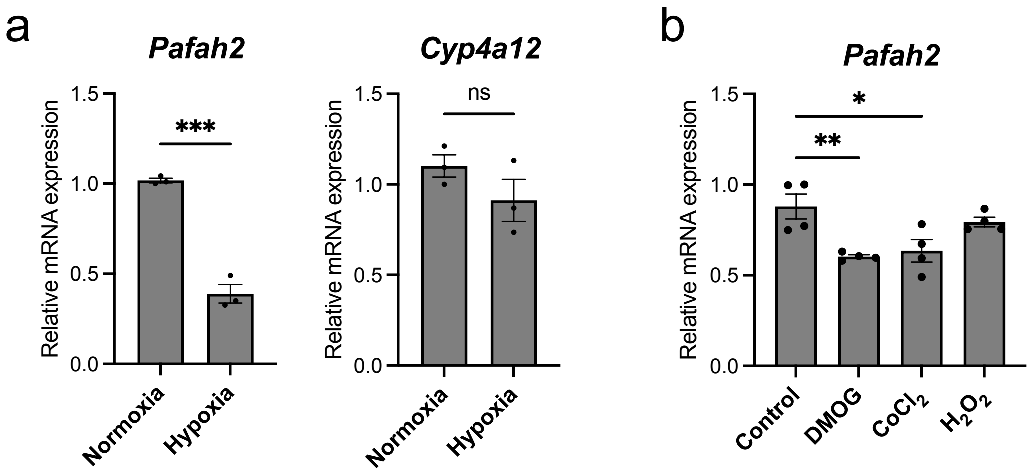

Supplementary Figure 9. (a) Relative mRNA levels of *Pafah2* and *Cyp4a12* in BMMCs exposed to normoxia or hypoxia (1% O_2) for 24 hours (n=3). Expression levels were normalized to those of 18S ribosomal RNA and then to those in BMMCs under normoxic condition. Data are representative of 3 independent experimental replicates. *** *P*=0.0003; ns indicates not significant. **(b)** Relative mRNA levels of *Pafah2* in BMMCs stimulated by DMOG (1mM), $CoCl_2$ (500µM), or H_2O_2 (500µM) for 6 hours (n=4). Expression levels were normalized to those of 18S ribosomal RNA and then to those in control BMMCs. Data are representative of 3 independent experimental replicates. * *P*=0.0100; ** *P*=0.0044. Data are mean ± SEM. *P* values were determined by 2-tailed Student's T test (a) or one-way ANOVA with Dunnett's *post hoc* test (b).

Supplementary Figure 10

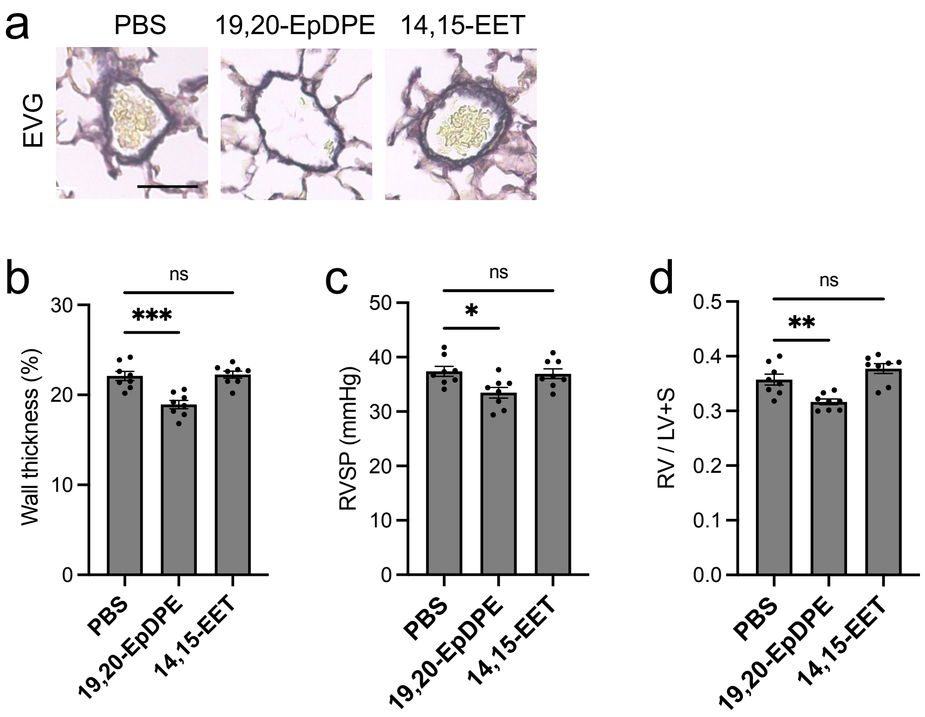

Supplementary Figure 10. (a) EVG staining in lungs of WT mice exposed to hypoxia for 4 weeks when administered PBS, 19,20-EpDPE (0.05 mg/kg/day), or 14,15-EET (0.05 mg/kg/day) i.p. every day. These administrations were started 2 weeks after hypoxic exposure. Scale bar, 50μm. **(b-d)** The evaluation of PH severity in WT mice exposed to hypoxia for 4 weeks when administered PBS, 19,20-EpDPE (0.05 mg/kg/day), or 14,15-EET (0.05 mg/kg/day) (n=8). Wall thickness of pulmonary arterioles (b), RVSP (c), weight ratio of RV to LV+septum (d). * P=0.0139; ** P=0.0043; *** P=0.0001. Data are mean ± SEM. ns indicates not significant. P values were determined by one-way ANOVA with Dunnett's *post hoc* test.

Supplementary Figure 11

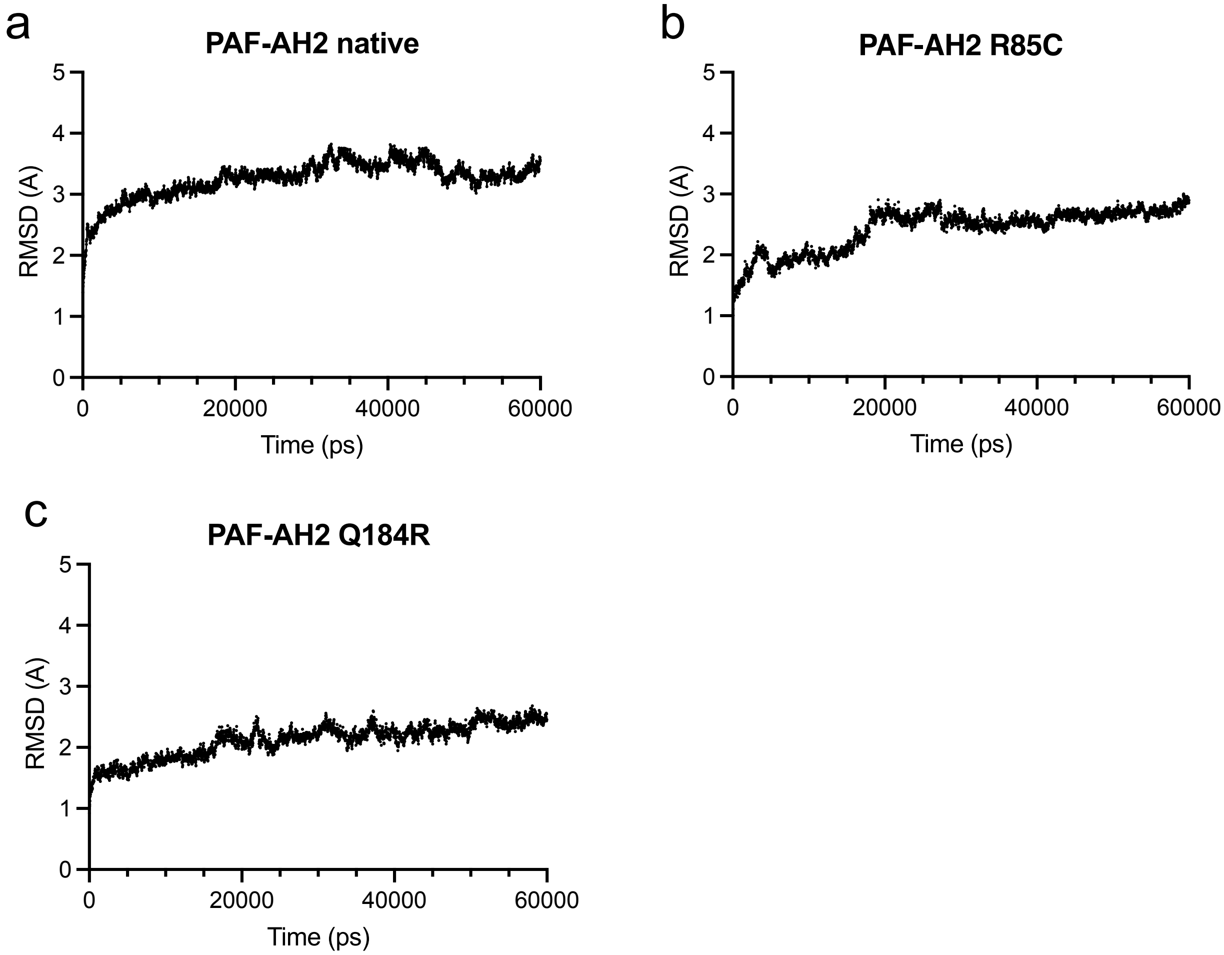

Supplementary Figure 11. (a-c) Root mean square deviation (RMSD) plot showing stabilization of the model structure in 60 nano-seconds. PAF-AH2 native (a), PAF-AH2 R85C variant (b), and PAF-AH2 Q184R variant (c).

SUPPLEMENTARY TABLES

Supplementary Table 1. Number of patients underwent whole-exome sequencing in each clinical classification of PH

Clinical classification		n
1	PAH	
1.1	Idiopathic PAH	90
1.2	Heritable PAH	
1.2.1	BMPR2	51
1.2.2	Other mutations	10
1.3	Drugs and toxin induced	6
1.4	Associated with:	
1.4.1	Connective tissue disease	54
1.4.2	HIV infection	1
1.4.4	Congenital heart diseases	30
1'	PVOD and/or pulmonary capillary hemangiomatosis	11
4	CTEPH and other pulmonary artery obstructions	5
5	PH with unclear and/or multifactorial mechanisms	4
Total		262

Patients were classified into clinical classification of PH according to ESC/ERS guideline 2015[1].

PAH, pulmonary arterial hypertension; BMPR2, bone morphogenetic protein receptor type II; HIV, human immunodeficiency virus; PVOD, pulmonary veno-occlusive disease; CTEPH, chronic thromboembolic pulmonary hypertension; PH, pulmonary hypertension.

Supplementary Table 2. Characteristics of patients with *Pafah2* variants and their clinical data.

Case	1	2	3
Diagnosis	idiopathic PAH	idiopathic PAH	borderline PH
Comorbidities	-	-	MCTD
Gender	female	female	female
Clinical data at diagnosis			
Age at diagnosis, years old	16	59	39
WHO-FC	2	2	2
Height, cm	147	149	158
Body weight, kg	40	56	58
Mean RAP, mmHg	5	2	6
Mean PAP, mmHg	45	41	21
Cardiac output, L/min	4.1	6.1	5.0
PVR, Wood units	8.5	6.4	1.6
PAWP, mmHg	10	2	13
BNP, pg/mL	21	106	45
6MWD, m	563	245	325
Clinical data at follow-up			
Medication	Tadalafil<br>Macitentan<br>Epoprostenol	Tadalafil<br>Macitentan	Sildenafil<br>Ambrisentan
Mean PAP, mmHg	41	34	16
PVR, Wood units	7.0	6.0	1.0
Pafah2 variant	c.253C>T<br>(p.Arg85Cys)	c.551A>G<br>(p.Gln184Arg)	c.253C>T<br>(p.Arg85Cys)
CADD PHRED*[1]	32.0	32.0	32.0
SIFT*[1]	deleterious	deleterious	deleterious
Polyphen*[1]	probably damaging	probably damaging	probably damaging
Total AF*[2]	0.0022	0.000863	0.0022
Japanese AF*[3]	0.0045	0	0.0045
Mutations in known PAH-related genes	-	*ATP13A3*,<br>c.736A>G<br>(p.Ile246Val)	-
CADD PHRED*[1]	-	23.2	-
SIFT*[1]	-	tolerated	-
Polyphen*[1]	-	possibly damaging	-
Total AF*[2]	-	0.0001125	-
Japanese AF*[3]	-	0.0001	-

PAH, pulmonary arterial hypertension; PH, pulmonary hypertension; MCTD, mixed connective tissue disease; WHO-FC, World Health Organization function class; RAP, right atrial pressure; PAP, pulmonary arterial pressure; PVR, pulmonary vascular resistance; PAWP, pulmonary arterial wedge pressure; BNP, B-type natriuretic peptide; 6MWD, 6-minute walk distance; AF, allele frequency.

*[1]; Pathogenicity scores were obtained from CADD web site (https://cadd.gs.washington.edu/).

*[2]; Allele frequencies were obtained from ExAc browser (Beta) (http://exac.broadinstitute.org/).

*[3]; Allele frequencies for Japanese general population were obtained from ToMMo 3.5KJSNV ver.1/2 data.

Supplementary Table 3. List of primer sequences used for real-time PCR

Mouse	Forward	Reverse
Nppa	ACCTGCACCACCTGGAGGAG	CCTTGGCTGTTATCTTCGGTACCG
Col1a1	CCTCAAGGGCTCCAACGAG	TCAATCACTGTCTTGCCCCA
Acta2	CGAAACCACCTATAACAGCATCA	GCGTTCTGGAGGGGCAAT
Il6	GTCCTTCAGAGAGATACAGAAACT	AGCTTATCTGTTAGGAGAGCATTG
Pafah2	GAAAGAGTGTGTGCGAGTGC	AGCAAGAACAGCCGTAGCTC
Cyp4a12	GCCTTATACGGAAATCATGGC	TGGAATCCTGGCCAACAATC
18S	CTTAGAGGGACAAGTGGCG	ACGCTGAGCCAGTCAGTGTA
Human	Forward	Reverse
Acta2	CTATGAGGGCTATGCCTTGCC	GCTCAGCCAGTAGTAACGAAGGA
Snail	TCGGAAGCCTAACTACACAGCGA	AGATGAGCATTGGCAGCGAG
Slug	CGAACTGGACACACATACAGTG	CTGAGGATCTCTGGTTGTGGT
Edn1	AGAGTGTGTCTACTTCTGCCA	CTTCCAAGTCCATACGGAACAA
Pdgfb	CTCGATCCGCTCCTTTGATGA	CGTTGGTGCGGTCTATGAG
Nos3	TGATGGCGAAGCGAGTGAAG	ACTCATCCATACACAGGACCC
Il1b	TTCGACACATGGGATAACGAGG	TTTTTGCTGTGAGTCCCGGAG
Tnf	TCAGATCATCTTCTCGAACCCC	ATCTCTCAGCTCCACGCCAT
18S	CTACCACATCCAAGGAAGCA	TTTTTCGTCACTACCTCCCCG

SUPPLEMENTARY REFERENCES

1. Galie N, *et al.* 2015 ESC/ERS Guidelines for the diagnosis and treatment of pulmonary hypertension: The Joint Task Force for the Diagnosis and Treatment of Pulmonary Hypertension of the European Society of Cardiology (ESC) and the European Respiratory Society (ERS): Endorsed by: Association for European Paediatric and Congenital Cardiology (AEPC), International Society for Heart and Lung Transplantation (ISHLT). *European heart journal* **37**, 67-119 (2016).
